# The efficacy of digital cognitive behavioral therapy for insomnia and depression: a systematic review and meta-analysis of randomized controlled trials

**DOI:** 10.7717/peerj.16137

**Published:** 2023-10-31

**Authors:** Wenyao Lin, Na Li, Lili Yang, Yuqing Zhang

**Affiliations:** 1 Department of Psychology, University of Chinese Academy of Sciences, Beijing, China; 2 Key Laboratory of Mental Health, Institute of Psychology, Chinese Academic of Sciences, Beijing, China

**Keywords:** Digital cognitive behavioral therapy for insomnia, Insomnia, Depression, Systematic review, Meta-analysis

## Abstract

**Background:**

Insomnia and depression often co-occur. Cognitive behavioral therapy for insomnia (CBT-I) seems to be effective and safe for mitigating insomnia and depression. However, the efficacy of digitally-delivered CBT-I (dCBT-I) remains unclear. Therefore, this meta-analysis of randomized controlled trials (RCTs) was to systematically review and evaluate the efficacy of dCBT-I in adults with insomnia and depression.

**Methods:**

A systematic search in PubMed, Cochrane, Embase, and Web of Science databases (as of June 5, 2022) was conducted for RCTs on dCBT-I. Statistical analyses were performed using Revan Manager. The effects of dCBT-I on insomnia and depression were expressed as standardized mean difference (SMD) with 95% confidence intervals (CIs).

**Results:**

Seven studies involving 3,597 participants were included. This meta-analysis showed that dCBT-I reduced the severity of insomnia (SMD = −0.85, 95% CI [−1.00 to −0.69], *p* < 0.001) and depression (SMD = −0.47, 95% CI [−0.55 to −0.38], *p* < 0.001) in short terms, and also mitigated the severity of insomnia (SMD = −0.71, 95% CI [−1.00 to −0.44], *p* < 0.001) and depression (SMD = −0.42, 95% CI [−0.68 to −0.15], *p* = 0.002) in long terms. The effect of dCBT-I was comparable to that of traditional face-to-face CBT-I, and was generally maintained at follow-ups of 6 weeks to 6 months.

**Conclusion:**

dCBT-I seems to be effective in alleviating insomnia and depression and might be considered as a viable treatment option for depression.

## Introduction

Insomnia is a complex and common disorder characterized by unsatisfactory sleep quality due to difficulties in initiating and/or maintaining sleep, with the global prevalence being about one-fifth of the general population (Zhang et al., 2019b; American Psychiatric Association, 2000; Seow et al., 2018). This ailment frequently co-occurs with mental health disorders, such as major depression, and is a risk factor for both incident and recurrent episodes of depression, imposing a critical public health problem worldwide (Ohayon, 2002). Up to 84%–90% of people with depression have reported sleep and circadian disturbances (Germain & Kupfer, 2008; Tsuno, Besset & K, 2005; Franzen & Buysse, 2008). Meanwhile, people with insomnia were 9.82 times more likely to have clinical depression and suffered from greater depression severity than those without insomnia (Taylor et al., 2005). Although the underlying pathophysiology of insomnia and depression remains elusive, a bidirectional relationship has been demonstrated between the two. Thus, treatments targeting both insomnia and depression might lead to larger and more sustained improvements than those for insomnia or depression alone (Franzen & Buysse, 2008; Smith, Huang & Manber, 2005).

Cognitive behavioral therapy for insomnia (CBT-I) is a non-pharmacological treatment that consists of multiple components, including cognitive therapy, stimulus control, sleep restriction, sleep hygiene, and relaxation (Edinger et al., 2021). It is recommended as the first-line therapy for patients with primary and comorbid insomnia (Van der Zweerde et al., 2019; Trauer et al., 2015). This approach has been demonstrated effective for relieving insomnia and sleep-related complaints in people with depression (Feng et al., 2020; Ballesio et al., 2018), as well as mitigating both incident and recurrent depression (Nakagawa et al., 2017; Hrynyschyn & Dockweiler, 2021; Lin, Lin & Chen, 2022). People receiving CBT-I have shown moderate-to-large, immediate, and sustained improvements in sleep quality and quantity (Van Straten et al., 2018; Mitchell et al., 2012). Despite its efficacy, the traditional face-to-face CBT-I was not widely accepted due to various limitations, such as the requirement of 6–8 weeks of direct contact, personal travel, and high costs (McCall, 2018; Koffel, Bramoweth & Ulmer, 2018). Given the imbalanced geographical distributions of CBT-I providers and a lack of certified therapists (Thomas et al., 2016), digitally-delivered CBT-I (dCBT-I) *via* web or mobile appears to be a more promising option compared to traditional face-to-face CBT-I (Molloy & Anderson, 2022; Hedman, Ljótsson & Lindefors, 2012), as it enables patients to receive CBT-I treatment from experienced therapists online.

Although dCBT-I is still nascent, the growing evidence and meta-analyses have corroborated its efficacy for insomnia (Van Straten et al., 2018; Ström, Pettersson & Andersson, 2004; Espie et al., 2012). However, RCTs on the efficacy of dCBT-I in alleviating depression are lacking (Horsch et al., 2017; Cheng et al., 2019). The dCBT-I approach may effectively mitigate insomnia and depression across a wide population. This systematic review and meta-analysis is to assess the efficacy of dCBT-I in individuals with insomnia and depression, providing clinical references for the management of insomnia and depression comorbidity.

## Materials & Methods

### Search strategy

The current study was carried out according to the Preferred Reporting Items for Systemic Reviews and Meta-Analysis criteria (PRISMA) guidelines (Page et al., 2021). The study protocol was registered in the International Prospective Register of Systematic Reviews (PROSPERO, http://www.crd.york.ac.uk/prospero), registration number: CRD42022355049. The methodology differs from the registered protocol on PROSPERO, because adjustments have been made during the review process. Between-group comparisons for depression symptoms (registered on PROSPERO) at post-treatment and follow-up time points were conducted, while the differences in insomnia severity at post-treatment and follow-up time points were calculated. The search strategy was designed by two researchers (Wenyao Lin and Na Li) using a combination of free words and relevant Mesh terms (dCBT-I, insomnia, and depression). The search strategy is detailed in Table S1. PubMed, Cochrane, Embase, and Web of Science databases were searched from 1 January 2000 to 5 June 2022. Grey literature, existing systematic reviews, and meta-analysis were also searched to identify potential studies which met the inclusion and exclusion criteria. Furthermore, we updated the search to collect relevant publications during 2022 and found two additional relevant articles (Wiklund et al., 2022; Mason et al., 2023). Due to the small amount of updated literature, we did not update the data analysis.

### Inclusion and exclusion criteria

The inclusion and exclusion criteria for primary studies were designed to select high-quality clinical trials, allowing for drawing reliable conclusions regarding the efficacy of dCBT-I in patients with insomnia and depression. The inclusion criteria followed the Participant, Intervention, Comparison, Outcome, and Study (PICOS) guidelines (Liberati et al., 2009). Studies meeting the following criteria were included in this meta-analysis:

#### Participant

Patients diagnosed with insomnia and depression, older than 18 years. The insomnia severity was measured by using the Insomnia Severity Index (ISI) (Bastien, Vallières & Morin, 2001) or the 5th-Diagnostic and Statistical Manual of mental disorders (DSM-5). Depression was diagnosed based on any recognized scales (*i.e.,* HADS, PHQ, and EPDS).

#### Intervention

The intervention group received dCBT-I treatment, including different forms, such as sleep restriction, stimulus control, relaxation training, sleep education and cognitive therapy;

#### Comparison

The control group received no treatment, placebo, or treatment as usual without specific sleep treatment;

#### Outcome

Sleep-/depression-related outcomes at baseline, post-treatment, and final follow-up in both treatment and control groups;

#### Study type

Randomized controlled trials (RCTs).

The following exclusion criteria were used: (1) Patients could not be diagnosed with insomnia; (2) studies that did not use a digital form of dCBT-I as the treatment, or intervention was not dCBT-I alone, such as dCBT-I + hypnotics; (3) depression symptoms were not reported; (4) comments, conference abstracts, letters, or systemic reviews; (5) non-English literature.

### Data extraction

Data were independently extracted from the included studies by two researchers (Wenyao Lin and Lili Yang). Any difference in the extracted data was resolved by discussion. After literature selection, the following information was manually extracted from all included studies: the first author, year of publication, sample size, age of participants, percentage of females, therapeutic components, treatment duration, diagnostic approach for insomnia and depression, mean and standard deviation for the severity of insomnia and depression at baseline, post-treatment, and final follow-up.

### Quality assessment

The methodological quality of the included studies was assessed using the Cochrane Collaboration risk of bias assessment tool (Higgins et al., 2011). Each study was evaluated from seven specific domains: random sequence generation, allocation concealment, blinding of participants and personnel, blinding of outcome assessment, incomplete outcome data, selective reporting, and other sources of bias (sample size estimation, complete definition of intervention sessions and outcomes in the intervention and control groups). Two researchers independently rated each domain as low (green), unclear (yellow), or high (red) risk of bias. If there were any dissents, the corresponding author was consulted.

### Statistical analysis

Statistical analyses were performed using Revan Manager (version 5.4). Outcomes of insomnia and depression were all continuous variables, and therefore standardized mean difference (SMD) was used to pool effect sizes. Effect sizes were calculated by subtracting the mean post-treatment/final follow-up score in the treatment group from the mean score in the control group, and dividing the result by the pooled standard deviation of the two groups. Using the post-treatment/follow-up mean score, instead of a change in mean scores between baseline and post-treatment/follow-up, is less likely to cause bias in effect sizes, and baseline differences do not necessarily lead to discrepancy in conclusions (Fu & Holmer, 2016). Effect sizes of 0.2, 0.5, and 0.8 are considered to be small, medium, and large respectively (Borenstein et al., 2010). Assuming that there are multiple true effect sizes rather than a single true effect size, a random-effects model (Borenstein et al., 2010) was adopted for data analysis. Statistical heterogeneity among included studies was evaluated with I^2^ statistics. The I^2^ values of 0% to 40%, 41% to 74%, and 75% to 100% represent low, medium, and considerable heterogeneity, respectively (Higgins & Green SJN-SAfePuP, 2011). All tests were two-sided, with *p* < 0.05 considered statistically significant.

### Sensitivity analysis and publication bias

Sensitivity analysis was performed to explore the source of heterogeneity. Publication bias was visually assessed using funnel plots and statistically examined using Egger’s tests (Egger, Smith & Phillips, 1997). The sensitivity analysis and publication bias test were performed using the STATA statistical software (version 15.0; College Station, TX, USA).

## Results

### Retrieval results and study characteristics

**Figure 1:**
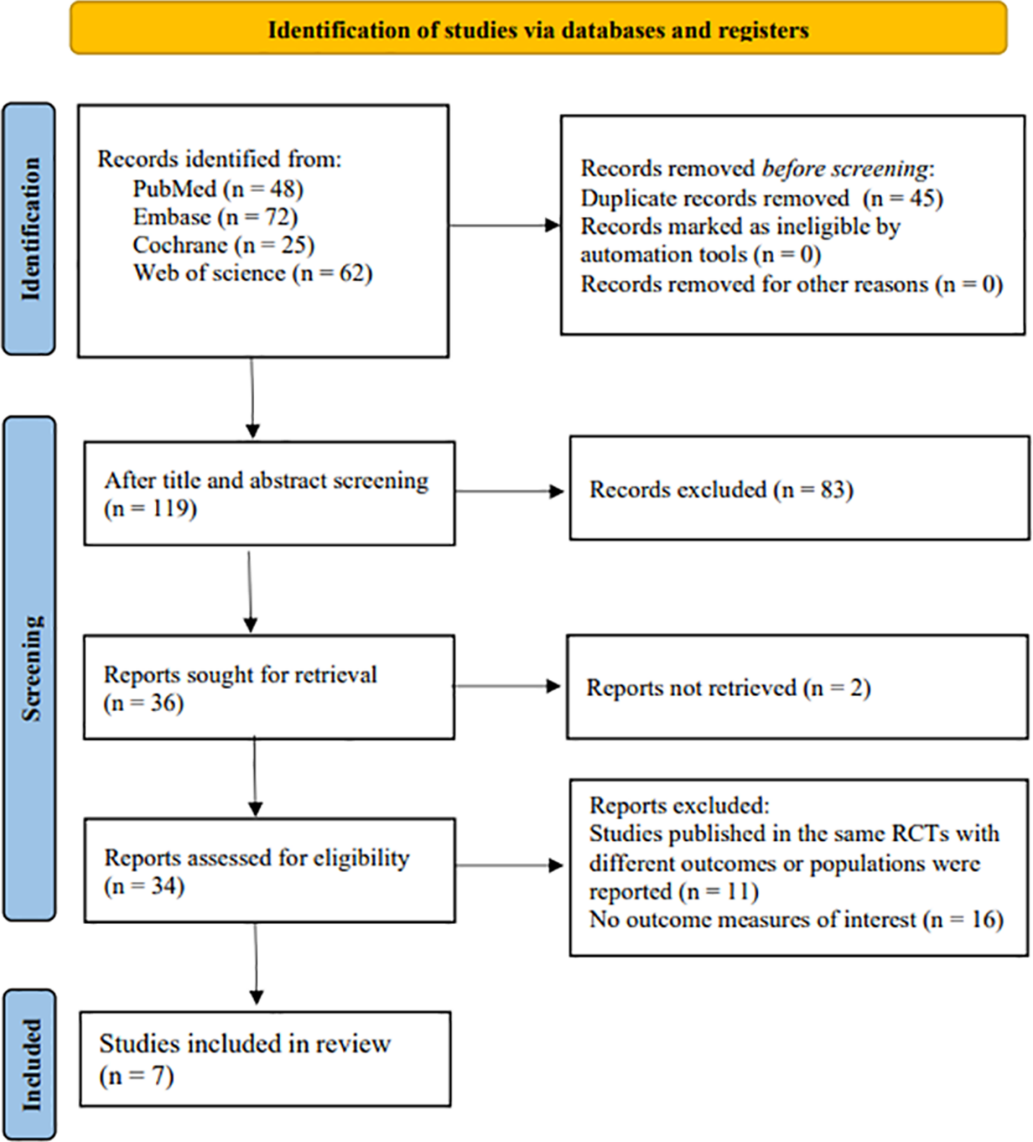
Study selection procedure.

A total of 207 records were identified in the database search. After duplicated records were removed, there were 162 records left. Upon the title and abstract screening, 83 ineligible records were excluded. Then, after the full texts of the remaining 119 records were read, 83 records that were not meet the inclusion criteria were deleted. Of the remaining 36 studies, seven articles were included in the present study. The literature screening process is shown in Fig. 1.

Of the included seven studies (Horsch et al., 2017; Cheng et al., 2019; Freeman et al., 2017; Rajabi Majd et al., 2020; Kyle et al., 2020; Faaland et al., 2022; Kalmbach et al., 2020), most were conducted in European regions, including UK (*n* = 2), Norway (*n* = 1) and Netherlands (*n* = 1). The remaining studies were carried out in USA (*n* = 2) or China (*n* = 1). The dCBT-I was delivered *via* mobile phone apps in three studies (*n* = 3) and *via* internet programs in other studies (*n* = 4). The diagnostic criteria for insomnia were based on the DSM-5 in two studies (Cheng et al., 2019; Kyle et al., 2020), the Insomnia Severity Index (ISI) in three studies (Horsch et al., 2017; Faaland et al., 2022; Kalmbach et al., 2020), both in one study (Rajabi Majd et al., 2020), and the Sleep Condition Indicator in one study (Freeman et al., 2017). The dCBT-I employed in all included studies was multi-component, including sleep hygiene education (SHE), cognitive restructuring (CR), sleep restriction (SC), and stimulus control (SC). The dCBT-I components differed between studies. The specific dCBT-I components in the included studies are shown in Table 1. In contrast, the program length was mostly similar. Six studies provided a 6-session dCBT-I program, while one study adjusted the dCBT-I program for each participant (Horsch et al., 2017). Most of the included studies were done within 12 weeks (1, 9, 10 or 12 weeks), while one study examined the effect of dCBT-I after 3 months of treatment (Horsch et al., 2017). Four studies reported the follow-up time, at 6 weeks (*n* = 1), 22 weeks (*n* = 1), and 24 weeks (*n* = 1), 6 months (*n* = 1), respectively. All included studies used the ISI to assess the severity of insomnia. At the same time, depression was measured using different scales, including Quick Inventory of Depression (QIDS) (Cheng et al., 2019), Hospital Anxiety and Depression scale (HADS) (Rajabi Majd et al., 2020; Faal et al., 2022), Patient Health Questionnaire (PHQ) (Freeman et al., 2017; Kyle et al., 2020), Edinburgh Postnatal Depression scale (EPDS) (Kalmbach et al., 2020), and Center of Epidemiological Studies Depression scale (CES-D) (Horsch et al., 2017).

**Table 1:** Characteristics and quality assessment of the included studies.

**Study**	**Country**	**Insomnia criteria**	**dCBT-I components**	**Duration**	**Insomnia**	**Depression**	**Post-treatment**	**Final follow-up**
Cheng et al. (2019)	USA	DSM-5	SHE-CR-SR-SC	12 W	ISI	QIDS	12W	–
Faaland et al. (2022)	Norway	ISI ≥ 12	SHE-CR-SR-SC-RP	9 W	ISI	HADS	9W	–
Freeman et al. (2017)	UK	SCI ≤16	SHE- CR-SR- SC-RLX	10W	ISI	PHQ	10W	22W
Kalmbach et al. (2020)	USA	ISI ≥ 10	SHE- CR-SR- SC-RLX	1W	ISI	EPDS	1W	6W
Kyle et al. (2020)	UK	DSM-5	SHE- CR-SR-SC-RLX	10W	ISI	PHQ	10W	24W
Rajabi Majd et al. (2020)	Iran	ISI >10; DSM-5	SHE-CR-SM-PS	6W	ISI	HADS	1M	6M
Horsch et al. (2017)	Netherlands	ISI ≥ 7	SHE-CR-SR-RLX	7W	ISI	CES-D	3M	–

**Notes.**

RCT randomized controlled trial SHE sleep hygiene education CR cognitive restructuring SR sleep restriction SC stimulus control RP relapse prevention SR self-monitoring PS problem solving ISI Insomnia Severity Index SCI Sleep Condition Indictor QIDS Quick Inventory of Depression HADS Hospital Anxiety and Depression scale PHQ Patient Health Questionnaire EPDS Edinburgh Postnatal Depression Scale CES-D center of epidemiological studies depression scale W weeks M months

There were 3,597 participants in the dCBT-I treatment group (mean age = 24.8–52.5, 1990 (55%) females) and 3,500 in the control group (mean age = 24.6–52.4, 1928 (55%) females). One study did not report age or gender. The smallest sample size was 101, and the largest was 3,755. The dropout rate in each study was calculated, with an average of 29.8% in the intervention group and 23.6% on the waiting list. Table 2 shows the basic characteristics of the included studies.

**Table 2:** Demographics of the included studies and rating scores.

**Study**	**Group**	**N**	**Age** mean (SD)	**Sex** **(F, %)**	**Insomnia**		**Depression**	
					**Pre-test**	**Post-test**	**Pre-test**	**Post-test**
Cheng et al. (2019)	TT	358	44.5 (15.8)	78%	17.9 (4.3)	7.9 (5.7)	10.8 (4.5)	6.7 (4.7)
	CT	300	45.7 (15.1)	80%	17.7 (4.4)	13.3 (4.6)	10.8 (4.6)	9.2 (3.7)
Faaland et al. (2022)	TT	270	–	–	19.2 (4.0)	10.5 (5.8)	13.5 (7.1)	10.5 (7.0)
	CT	262	–	–	19.5 (4.0)	15.2 (5.3)	13.6 (7.3)	11.8 (7.0)
Freeman et al. (2017)	TT	1891	24.8 (7.7)	72%	15.4 (3.9)	9.2 (5.2)	12.9 (5.8)	8.4 (6.2)
	CT	1864	24.6 (7.6)	71%	15.3 (4.0)	13.0 (5.3)	12.7 (5.9)	11.3 (6.7)
Kalmbach et al. (2020)	TT	46	28.9 (28.9)	100%	14.9 (3.6)	10.0 (5.7)	9.5 (4.6)	5.9 (4.3)
	CT	45	29.2 (4.4)	100%	14.1 (3.4)	12.9 (4.8)	7.5 (4.1)	5.9 (5.3)
Kyle et al. (2020)	TT	205	52.5 (11.2)	85%	18.4 (3.7)	10.7 (5.1)	11.9 (5.4)	7.7 (6.3)
	CT	205	52.4 (11.7)	88%	17.9 (3.6)	16.2 (4.3)	11.4 (5.1)	10.9 (4.9)
Rajabi Majd et al. (2020)	TT	156	36.2 (5.8)	54%	19.3 (4.6)	12.7 (5.6)	6.3 (3.8)	3.8 (0.9)
	CT	156	35.3 (2.8)	58%	19.2 (4.6)	16.7 (5.0)	6.0 (3.3)	6.0 (3.1)
Horsch et al. (2017)	TT	74	39.0 (13.0)	61%	16.4 (3.3)	9.9 (4.9)	16.5 (6.0)	11.0 (5.6)
	CT	77	41.0 (13.9)	64%	16.4 (3.1)	13.2 (4.5)	15.0 (5.8)	15.5 (5.9)

**Notes.**

TT treatment group CT control group M Morning chronotype of insomnia I Intermediate chronotype of insomnia E Evening chronotype of insomnia

### Quality assessment

The Cochrane Collaboration risk of bias assessment tool was used to assess the quality of the included studies. Most studies used random sequence generation, had no selective reporting, and completely defined intervention sessions and outcomes in both intervention and control groups. Allocation concealment and blinding of participants and personnel were performed in most of the studies. More than half of the included studies did not report whether the outcome assessment was blinding. The quality assessment of the included studies is presented in Figs. S1 and S2.

### Short-term and long-term effects of dCBT-I on insomnia

**Figure 2:**
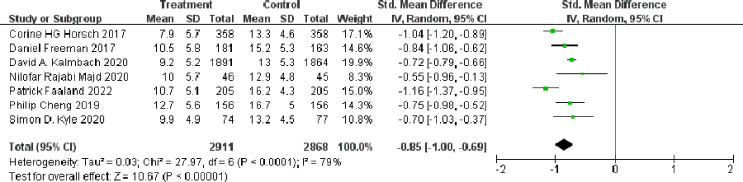
Forest plot of the short-term effect of dCBT-I on insomnia at post-treatment. Abbreviations: SD, standard deviation; Total, number of participants; IV, inverse variance; CI, confidence intervals (Horsch et al., 2017; Freeman et al., 2017; Kalmbach et al., 2020; Rajabi Majd et al., 2020; Faaland et al., 2022; Cheng et al., 2019; Kyle et al., 2020).

The self-reported ISI was provided in all eligible studies (Gullickson & Ramser, 1996), involving 5,779 participants (*n* = 2911 in the treatment group and *n* = 2868 in the control group). The effect size (Hedges’g) at post-treatment was calculated to quantify the short-term effect of dCBT-I on insomnia. The meta-analysis showed that dCBT-I significantly improved insomnia at post-treatment (SMD = −0.85, 95% CI [−1.00 to −0.69], *p* < 0.001) (Fig. 2).

**Figure 3:**
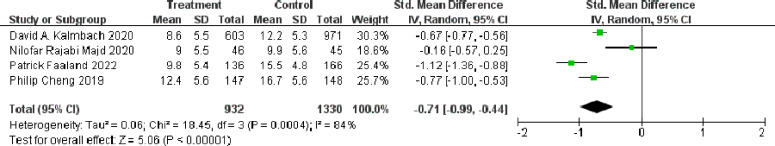
Forest plot of the long-term effect of dCBT-I on insomnia at final follow-up. Abbreviations: SD, standard deviation; Total, number of participants; IV, inverse variance; CI, confidence intervals (Kalmbach et al., 2020; Rajabi Majd et al., 2020; Faaland et al., 2022; Cheng et al., 2019).

Four studies conducted follow-up evaluations and therefore included in the meta-analysis of the long-term effect of dCBT-I on insomnia. In total, 932 participants in the treatment group and 1,330 in the control group completed the final follow-up evaluations (ranging from six weeks to six months). The dCBT-I also showed a significant improvement in insomnia at final follow-up (SMD = −0.71, 95% CI [−1.00 to −0.44], *p* < 0.001). More details of the effect of dCBT-I on insomnia at final follow-up are shown in Fig. 3.

### Short-term and long-term effects of dCBT-I on depression

**Figure 4:**
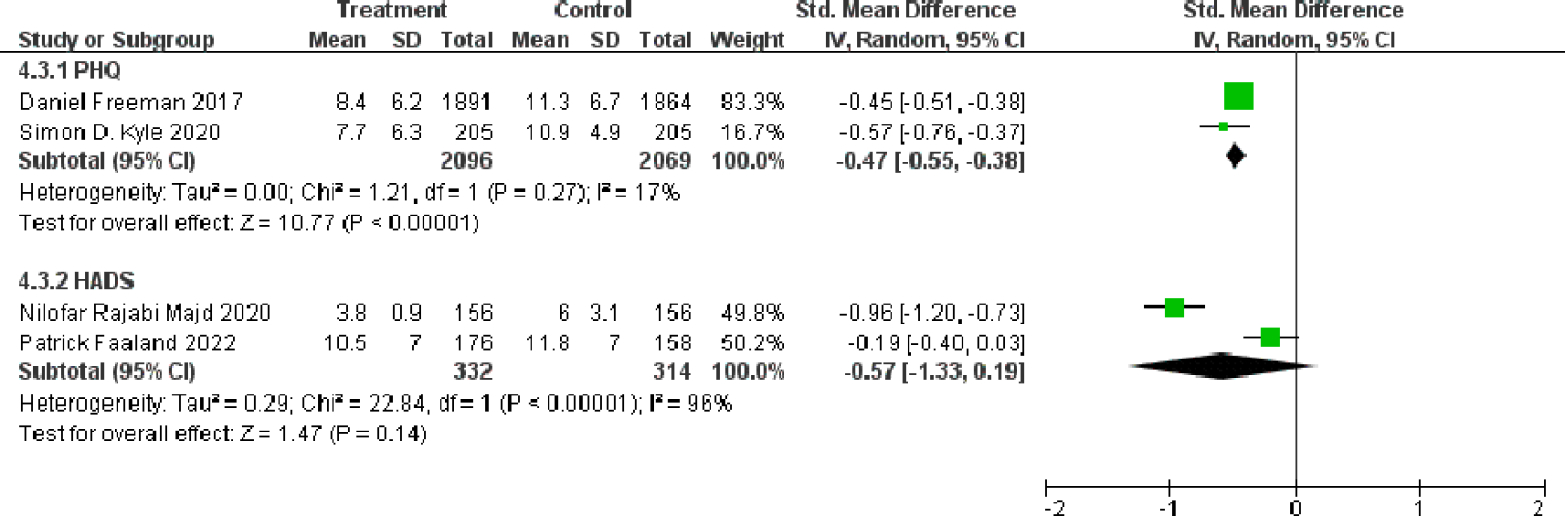
Forest plot of the short-term effect of dCBT-I on depression at post-treatment. Abbreviations: SD, standard deviation; Total, number of participants; IV, inverse variance; CI, confidence intervals (Freeman et al., 2017; Rajabi Majd et al., 2020; Faaland et al., 2022; Kyle et al., 2020).

As depression was measured using different scales, only included four studies were included: two studies measured depression with PHQ (Freeman et al., 2017; Kyle et al., 2020) and two studies measured depression with HADS (Rajabi Majd et al., 2020; Faal et al., 2022). Other three studies used QIDS (Cheng et al., 2019), EPDS (Kalmbach et al., 2020), and CES-D (Horsch et al., 2017) respectively. The results of meta-analyses showed that dCBT-I significantly reduced the PHQ score compared to the control group (SMD = −0.47, 95% CI [−0.55 to −0.38]), *p* < 0.001). However, there was no significant difference in the HADS score between the dCBT-I treatment group and the control group (SMD = −0.57, 95% CI [−1.33 to 0.19], *p* < 0.001) (Fig. 4).

**Figure 5:**
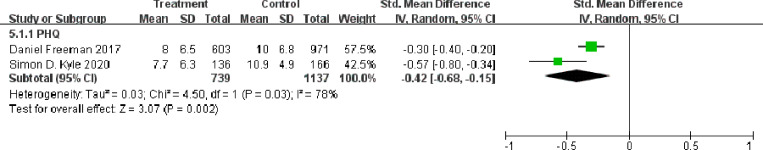
Forest plot of the long-term effect of dCBT-I on depression at final follow-up. Abbreviations: SD, standard deviation; Total, number of participants; IV, inverse variance; CI, confidence intervals (Freeman et al., 2017; Kyle et al., 2020).

The effect of dCBT-I on depression at final follow-up is shown in Fig. 5. dCBT-I also resulted in a statistically significant decrease in the PHQ score at the final follow-up (SMD = −0.42, 95% CI [−0.68 to −0.15], *p* = 0.002).

### Sensitivity analysis and publication bias

Sensitivity analysis showed that the meta-analysis results were relatively stable for insomnia (Fig. S3). Despite high heterogeneity in the ISI for the post-treatment (I^2^ = 79%) and final follow-up (I^2^ = 84%), Egger’s test showed no publication bias (post-treatment, *p* = 0.449; final follow-up, *p* = 0.974). The funnel plot for the ISI was nearly symmetrical, as presented in Fig. S4. As there were only 2 RCTs included in the meta-analysis of PHQ score and HADS score to evaluate the effect of dCBT-I on depression, respectively, we could not make an inverted funnel plot to assess the influence of publication bias on included studies.

## Discussion

This meta-analysis assessed the efficacy of dCBT-I in adults with insomnia and depression based on a small number of eligible RCTs (*n* = 7, as of 5 June 2022). The meta-analysis results revealed that dCBT-I was effective in alleviating insomnia and depression [SMD: short-term effect on insomnia, −0.85 (−1.00, −0.69) and depression, −0.47 (−0.55, −0.38); long-term effect on insomnia, −0.71 (−0.99, −0.44) and depression −0.42 (−0.68, −0.15). Thus, dCBT-I appears to be a viable alternative to the traditional CBT-I treatment for insomnia and depression (Van der Zweerde et al., 2019; Zhang et al., 2019a; Oar, Johnco & Ollendick, 2017).

dCBT-I, as an internet-based approach, allows individuals to communicate with their therapists at any time and any place, and it is less costly and more accessible compared to traditional face-to-face CBT-I (Molloy & Anderson, 2022; Hedman, Ljótsson & Lindefors, 2012). This approach can provide a feasible way to broaden access to CBT-I, especially for those who live in a remote area. Importantly, the dCBT-I approach can help individuals with insomnia learn how to manage themselves in case of recurrence (Karyotaki et al., 2021; Wogan et al., 2021). According to the present meta-analysis of the effect of dCBT-I on insomnia, the dCBT-I treatment was effective in improving insomnia in both the short and long terms. Besides, results also indicated that the dCBT-I treatment had a statistically significant effect on depression at post-treatment. Although only two studies reported the final follow-up evaluations for depression, our analysis also showed that the effects were maintained. These results were consistent with previous meta-analysis studies (Karyotaki et al., 2021; Zachariae et al., 2016; Frase et al., 2020). Our findings provide evidence-based support for the short- and long-term effects of dCBT-I on insomnia and depression comorbidity, highlighting the importance of an insomnia-specific treatment for patients with depression.

In this meta-analysis, the effect sizes for insomnia severity were generally larger and remained significant at final follow-up. However, in terms of depression symptoms, the effect sizes for HADS scores failed to reach significance, whereas the effect sizes for PHQ were statistically significant at post-treatment and final follow-up time points. A possible reason is the small number of eligible RCTs. Effect sizes for both insomnia and depression decreased from post-treatment to final follow-up, which is common, possibly because patients may fail to adhere to their treatment strategies with time passing (Hertenstein et al., 2022).

The risk of bias assessment showed that most included studies rigorously reported participants’ outcome and follow-up without selective reporting. Nevertheless, allocation concealment and blinding were commonly unreported, leading to a high risk of bias, probably due to the behavioral nature of the treatment. Although strict inclusion criteria were used to minimize the heterogeneity of included studies, the degree of statistical heterogeneity was high in the short- and long-term efficacy for insomnia. High heterogeneity might be attributed to the different components of dCBT-I treatment in the included studies, which may have different effects on insomnia and depression.

The present study has several limitations. First, the components of dCBT-I were diverse across the included studies, which may impact the effects of dCBT-I on insomnia and depression. Second, conclusions on depression may be biased due to the small number of eligible RCTs. Further studies on dCBT-I treatment for depression are needed. Third, the included studies used different scales (*e.g.*, HADS, QIDS, and PHQ) to assess the severity of depression, which may lead to premature conclusions. Further RCTs are needed to verify our findings.

## Conclusion

The dCBT-I approach was effective in alleviating insomnia and depression comorbidity. Further research should focus on the effect of diverse dCBT-I components and standardized sleep- and depression-related outcomes. Meanwhile, additional RCTs are also needed to corroborate the clinical efficacy of dCBT-I for patients with insomnia and depression.

## Supplemental Information

10.7717/peerj.16137/supp-1 Supplemental Information 1 Risk of bias summary: review of authors’ judgement on each risk of bias item for each included study

10.7717/peerj.16137/supp-2 Supplemental Information 2 Risk of bias graph: review of authors’ judgement on each risk of bias item, presented as percentages across all included studies

10.7717/peerj.16137/supp-3 Supplemental Information 3 Sensitivity analysis displaying the overall estimated pooled effect size of DCBT-I on insomnia at the post-treatment (a) and final follow-up (b) time points

10.7717/peerj.16137/supp-4 Supplemental Information 4 Funnel plot displaying the probable publication bias in estimated pooled effect size of dCBT-I on insomnia at the post-treatment (a) and final follow-up (b) time points

10.7717/peerj.16137/supp-5 Supplemental Information 5 Literature search strategy

10.7717/peerj.16137/supp-6 Supplemental Information 6 PRISMA checklist

10.7717/peerj.16137/supp-7 Supplemental Information 7 Systematic review and/or meta-analysis rationale

## Additional Information and Declarations

### Data Availability

The following information was supplied regarding data availability: This is a Systematic Review and/or Meta-Analysis Rationale.
