# Supplementary figures and images for: The efficacy of digital cognitive behavioral therapy for insomnia and depression: a systematic review and meta-analysis of randomized controlled trials
Source: PeerJ. 2023 Oct 31;11:e16137. doi: 10.7717/peerj.16137 (PMC10624170; doi:10.7717/peerj.16137)

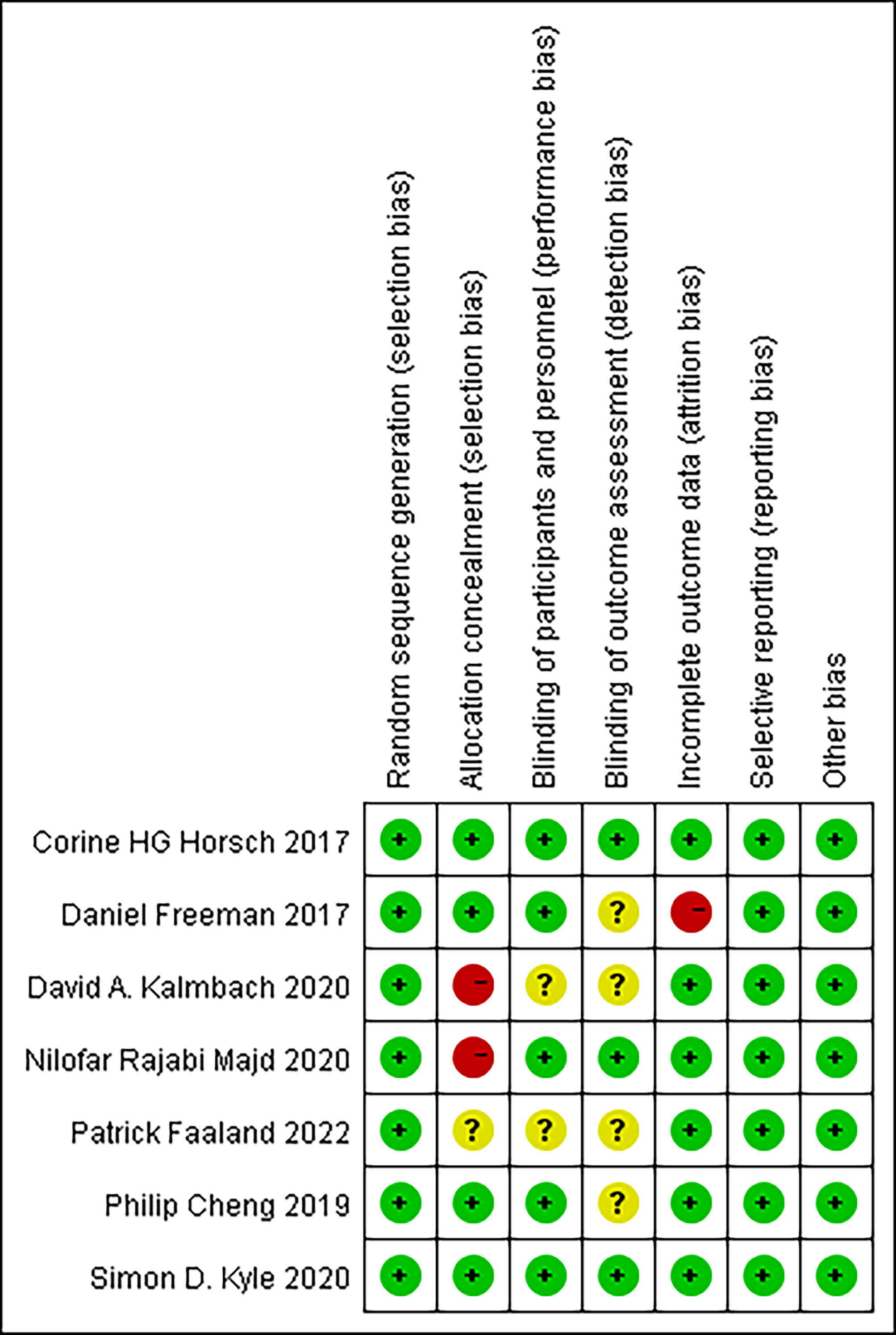

Supplement: Supplemental Information 1 [file peerj-11-16137-s001.png]

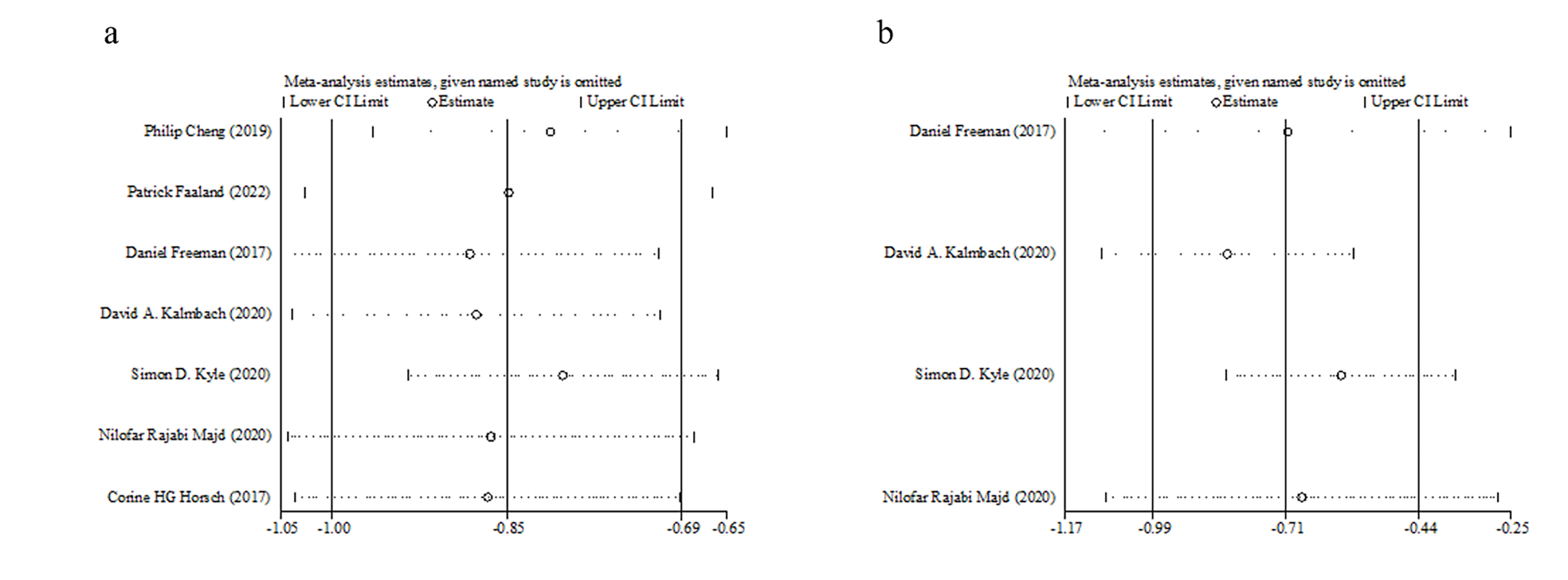

Supplement: Supplemental Information 3 [file peerj-11-16137-s003.png]

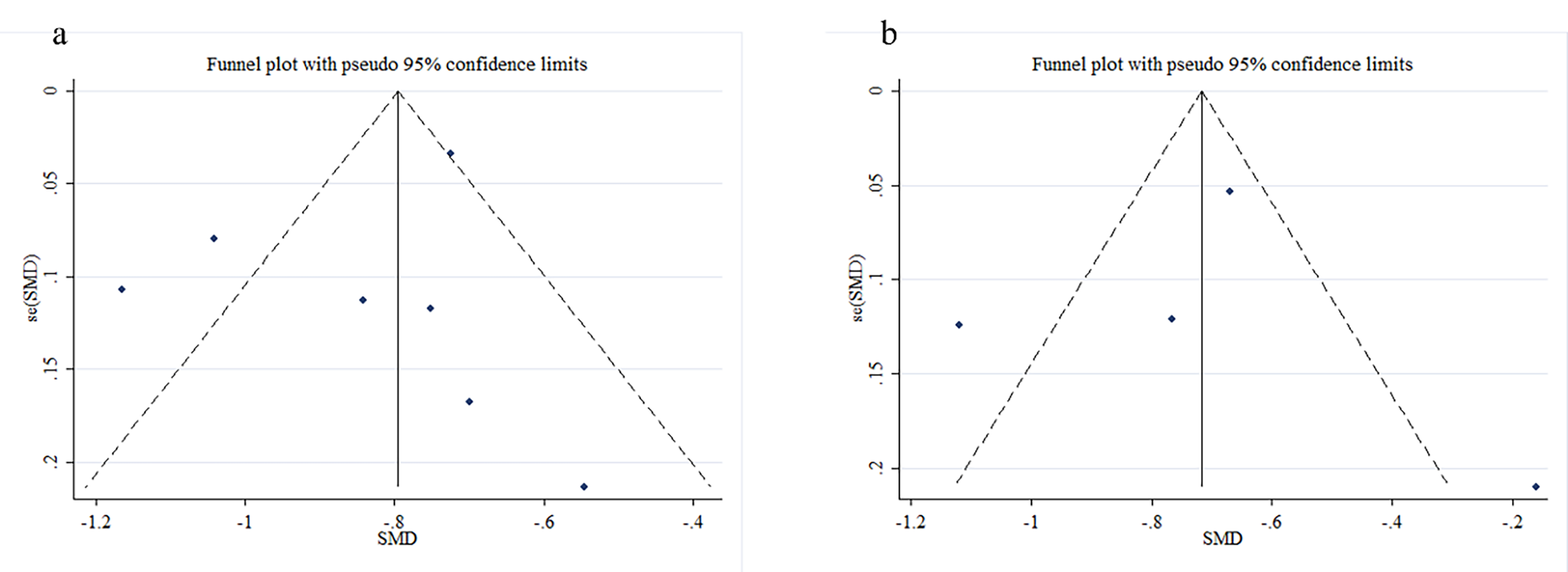

Supplement: Supplemental Information 4 [file peerj-11-16137-s004.png]
