# Supplementary material for: The efficacy of digital cognitive behavioral therapy for insomnia and depression: a systematic review and meta-analysis of randomized controlled trials
Source: PeerJ. 2023 Oct 31;11:e16137. doi: 10.7717/peerj.16137 (PMC10624170; doi:10.7717/peerj.16137)
Supplement: Supplemental Information 2 [file peerj-11-16137-s002.pdf]

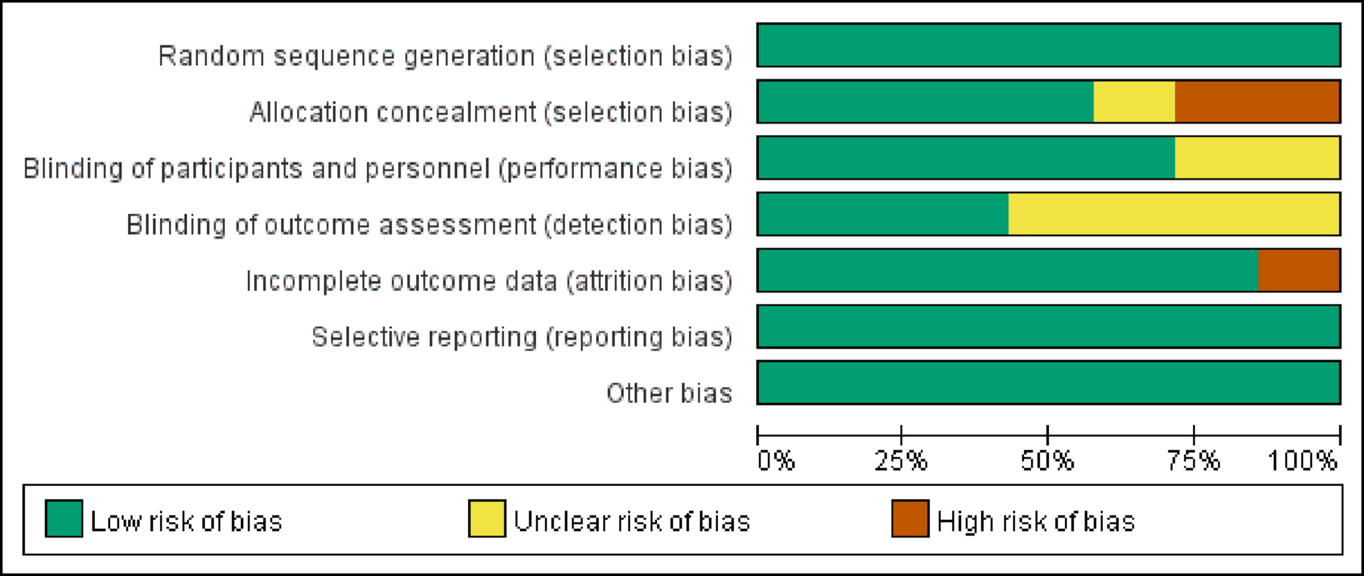
Random sequence generation (selection bias)
Allocation concealment (selection bias)
Blinding of participants and personnel (performance bias)
Blinding of outcome assessment (detection bias)
Incomplete outcome data (attrition bias)
Selective reporting (reporting bias)
Other bias
0%
25%
50%
75%
100%
Low risk of bias
Unclear risk of bias
High risk of bias
