# Supplementary material for: The efficacy of digital cognitive behavioral therapy for insomnia and depression: a systematic review and meta-analysis of randomized controlled trials
Source: PeerJ. 2023 Oct 31;11:e16137. doi: 10.7717/peerj.16137 (PMC10624170; doi:10.7717/peerj.16137)
Supplement: Supplemental Information 5 [file peerj-11-16137-s005.docx]

**Table S1** Literature search strategy

| Search number | Query |
| --- | --- |
| **1.Pubmed** | |
| #1 | "Sleep Initiation and Maintenance Disorders"[Mesh] |
| #2 | ((((((((((Sleep Initiation[Title/Abstract] AND Maintenance Disorders[Title/Abstract]) OR ("Disorders of Initiating and Maintaining Sleep"[Title/Abstract])) OR (Early Awakening[Title/Abstract])) OR (Awakening, Early[Title/Abstract])) OR (Sleep Initiation Dysfunction[Title/Abstract])) OR (Dysfunction, Sleep Initiation[Title/Abstract])) OR (Dysfunctions, Sleep Initiation[Title/Abstract])) OR (Sleep Initiation Dysfunctions[Title/Abstract])) OR (Sleeplessness[Title/Abstract])) OR (Insomnia[Title/Abstract])) OR (Insomnias[Title/Abstract]) |
| #3 | ("Sleep Initiation and Maintenance Disorders"[Mesh]) OR (((((((((((Sleep Initiation[Title/Abstract] AND Maintenance Disorders[Title/Abstract]) OR ("Disorders of Initiating and Maintaining Sleep"[Title/Abstract])) OR (Early Awakening[Title/Abstract])) OR (Awakening, Early[Title/Abstract])) OR (Sleep Initiation Dysfunction[Title/Abstract])) OR (Dysfunction, Sleep Initiation[Title/Abstract])) OR (Dysfunctions, Sleep Initiation[Title/Abstract])) OR (Sleep Initiation Dysfunctions[Title/Abstract])) OR (Sleeplessness[Title/Abstract])) OR (Insomnia[Title/Abstract])) OR (Insomnias[Title/Abstract])) |
| #4 | "Cognitive Behavioral Therapy"[Mesh] |
| #5 | ((((((((((((((((((((((((((((((Cognitive behavioral therapy[Title/Abstract]) OR (CBT[Title/Abstract])) OR (Behavioral Therapies, Cognitive[Title/Abstract])) OR (Behavioral Therapy, Cognitive[Title/Abstract])) OR (Cognitive Behavioral Therapies[Title/Abstract])) OR (Therapies, Cognitive Behavioral[Title/Abstract])) OR (Therapy, Cognitive Behavioral[Title/Abstract])) OR (Psychotherapy, Cognitive[Title/Abstract])) OR (Therapy, Cognitive[Title/Abstract])) OR (Cognitive Therapies[Title/Abstract])) OR (Therapies, Cognitive[Title/Abstract])) OR (Cognitive Therapy[Title/Abstract])) OR (Cognitive Behaviour Therapy[Title/Abstract])) OR (Behaviour Therapies, Cognitive[Title/Abstract])) OR (Behaviour Therapy, Cognitive[Title/Abstract])) OR (Cognitive Behaviour Therapies[Title/Abstract])) OR (Therapies, Cognitive Behaviour[Title/Abstract])) OR (Therapy, Cognitive Behaviour[Title/Abstract])) OR (Cognitive Psychotherapy[Title/Abstract])) OR (Cognitive Psychotherapies[Title/Abstract])) OR (Psychotherapies, Cognitive[Title/Abstract])) OR (Cognition Therapy[Title/Abstract])) OR (Cognition Therapies[Title/Abstract])) OR (Therapies, Cognition[Title/Abstract])) OR (Therapy, Cognitive Behavior[Title/Abstract])) OR (Behavior Therapies, Cognitive[Title/Abstract])) OR (Cognitive Behavior Therapies[Title/Abstract])) OR (Therapies, Cognitive Behavior[Title/Abstract])) OR (Therapy, Cognition[Title/Abstract])) OR (Behavior Therapy, Cognitive[Title/Abstract])) OR (Cognitive Behavior Therapy[Title/Abstract]) |
| #6 | ("Cognitive Behavioral Therapy"[Mesh]) OR (((((((((((((((((((((((((((((((Cognitive behavioral therapy[Title/Abstract]) OR (CBT[Title/Abstract])) OR (Behavioral Therapies, Cognitive[Title/Abstract])) OR (Behavioral Therapy, Cognitive[Title/Abstract])) OR (Cognitive Behavioral Therapies[Title/Abstract])) OR (Therapies, Cognitive Behavioral[Title/Abstract])) OR (Therapy, Cognitive Behavioral[Title/Abstract])) OR (Psychotherapy, Cognitive[Title/Abstract])) OR (Therapy, Cognitive[Title/Abstract])) OR (Cognitive Therapies[Title/Abstract])) OR (Therapies, Cognitive[Title/Abstract])) OR (Cognitive Therapy[Title/Abstract])) OR (Cognitive Behaviour Therapy[Title/Abstract])) OR (Behaviour Therapies, Cognitive[Title/Abstract])) OR (Behaviour Therapy, Cognitive[Title/Abstract])) OR (Cognitive Behaviour Therapies[Title/Abstract])) OR (Therapies, Cognitive Behaviour[Title/Abstract])) OR (Therapy, Cognitive Behaviour[Title/Abstract])) OR (Cognitive Psychotherapy[Title/Abstract])) OR (Cognitive Psychotherapies[Title/Abstract])) OR (Psychotherapies, Cognitive[Title/Abstract])) OR (Cognition Therapy[Title/Abstract])) OR (Cognition Therapies[Title/Abstract])) OR (Therapies, Cognition[Title/Abstract])) OR (Therapy, Cognitive Behavior[Title/Abstract])) OR (Behavior Therapies, Cognitive[Title/Abstract])) OR (Cognitive Behavior Therapies[Title/Abstract])) OR (Therapies, Cognitive Behavior[Title/Abstract])) OR (Therapy, Cognition[Title/Abstract])) OR (Behavior Therapy, Cognitive[Title/Abstract])) OR (Cognitive Behavior Therapy[Title/Abstract])) |
| #7 | "Mobile Applications"[Mesh] |
| #8 | (((((((((((((((((((((((((((((Mobile Applications[Title/Abstract]) OR (Application, Mobile[Title/Abstract])) OR (Applications, Mobile[Title/Abstract])) OR (Mobile Application[Title/Abstract])) OR (Mobile Apps[Title/Abstract])) OR (App, Mobile[Title/Abstract])) OR (Apps, Mobile[Title/Abstract])) OR (Mobile App[Title/Abstract])) OR (Portable Software Apps[Title/Abstract])) OR (App, Portable Software[Title/Abstract])) OR (Portable Software App[Title/Abstract])) OR (Software App, Portable[Title/Abstract])) OR (Portable Software Applications[Title/Abstract])) OR (Application, Portable Software[Title/Abstract])) OR (Portable Software Application[Title/Abstract])) OR (Software Application, Portable[Title/Abstract])) OR (Portable Electronic Apps[Title/Abstract])) OR (App, Portable Electronic[Title/Abstract])) OR (Electronic App, Portable[Title/Abstract])) OR (Portable Electronic App[Title/Abstract])) OR (Portable Electronic Applications[Title/Abstract])) OR (Application, Portable Electronic[Title/Abstract])) OR (Electronic Application, Portable[Title/Abstract])) OR (Portable Electronic Application[Title/Abstract])) OR (smartphone[Title/Abstract])) OR (Smartphones[Title/Abstract])) OR (Smart Phones[Title/Abstract])) OR (Smart Phone[Title/Abstract])) OR (Phones, Smart[Title/Abstract])) OR (Digital[Title/Abstract]) |
| #9 | ("Mobile Applications"[Mesh]) OR ((((((((((((((((((((((((((((((Mobile Applications[Title/Abstract]) OR (Application, Mobile[Title/Abstract])) OR (Applications, Mobile[Title/Abstract])) OR (Mobile Application[Title/Abstract])) OR (Mobile Apps[Title/Abstract])) OR (App, Mobile[Title/Abstract])) OR (Apps, Mobile[Title/Abstract])) OR (Mobile App[Title/Abstract])) OR (Portable Software Apps[Title/Abstract])) OR (App, Portable Software[Title/Abstract])) OR (Portable Software App[Title/Abstract])) OR (Software App, Portable[Title/Abstract])) OR (Portable Software Applications[Title/Abstract])) OR (Application, Portable Software[Title/Abstract])) OR (Portable Software Application[Title/Abstract])) OR (Software Application, Portable[Title/Abstract])) OR (Portable Electronic Apps[Title/Abstract])) OR (App, Portable Electronic[Title/Abstract])) OR (Electronic App, Portable[Title/Abstract])) OR (Portable Electronic App[Title/Abstract])) OR (Portable Electronic Applications[Title/Abstract])) OR (Application, Portable Electronic[Title/Abstract])) OR (Electronic Application, Portable[Title/Abstract])) OR (Portable Electronic Application[Title/Abstract])) OR (smartphone[Title/Abstract])) OR (Smartphones[Title/Abstract])) OR (Smart Phones[Title/Abstract])) OR (Smart Phone[Title/Abstract])) OR (Phones, Smart[Title/Abstract])) OR (Digital[Title/Abstract])) |
| #10 | "Depressive Disorder"[Mesh] |
| #11 | (((((((((((((((((((((((((Depressive Disorder[Title/Abstract]) OR (Depressive Disorders[Title/Abstract])) OR (Disorder, Depressive[Title/Abstract])) OR (Disorders, Depressive[Title/Abstract])) OR (Neurosis, Depressive[Title/Abstract])) OR (Depressive Neuroses[Title/Abstract])) OR (Depressive Neurosis[Title/Abstract])) OR (Neuroses, Depressive[Title/Abstract])) OR (Depression, Endogenous[Title/Abstract])) OR (Depressions, Endogenous[Title/Abstract])) OR (Endogenous Depression[Title/Abstract])) OR (Endogenous Depressions[Title/Abstract])) OR (Depressive Syndrome[Title/Abstract])) OR (Depressive Syndromes[Title/Abstract])) OR (Syndrome, Depressive[Title/Abstract])) OR (Syndromes, Depressive[Title/Abstract])) OR (Depression, Neurotic[Title/Abstract])) OR (Depressions, Neurotic[Title/Abstract])) OR (Neurotic Depression[Title/Abstract])) OR (Neurotic Depressions[Title/Abstract])) OR (Melancholia[Title/Abstract])) OR (Melancholias[Title/Abstract])) OR (Unipolar Depression[Title/Abstract])) OR (Depression, Unipolar[Title/Abstract])) OR (Depressions, Unipolar[Title/Abstract])) OR (Unipolar Depressions[Title/Abstract]) |
| #12 | ("Depressive Disorder"[Mesh]) OR ((((((((((((((((((((((((((Depressive Disorder[Title/Abstract]) OR (Depressive Disorders[Title/Abstract])) OR (Disorder, Depressive[Title/Abstract])) OR (Disorders, Depressive[Title/Abstract])) OR (Neurosis, Depressive[Title/Abstract])) OR (Depressive Neuroses[Title/Abstract])) OR (Depressive Neurosis[Title/Abstract])) OR (Neuroses, Depressive[Title/Abstract])) OR (Depression, Endogenous[Title/Abstract])) OR (Depressions, Endogenous[Title/Abstract])) OR (Endogenous Depression[Title/Abstract])) OR (Endogenous Depressions[Title/Abstract])) OR (Depressive Syndrome[Title/Abstract])) OR (Depressive Syndromes[Title/Abstract])) OR (Syndrome, Depressive[Title/Abstract])) OR (Syndromes, Depressive[Title/Abstract])) OR (Depression, Neurotic[Title/Abstract])) OR (Depressions, Neurotic[Title/Abstract])) OR (Neurotic Depression[Title/Abstract])) OR (Neurotic Depressions[Title/Abstract])) OR (Melancholia[Title/Abstract])) OR (Melancholias[Title/Abstract])) OR (Unipolar Depression[Title/Abstract])) OR (Depression, Unipolar[Title/Abstract])) OR (Depressions, Unipolar[Title/Abstract])) OR (Unipolar Depressions[Title/Abstract])) |
| #13 | (((("Sleep Initiation and Maintenance Disorders"[Mesh]) OR (((((((((((Sleep Initiation[Title/Abstract] AND Maintenance Disorders[Title/Abstract]) OR ("Disorders of Initiating and Maintaining Sleep"[Title/Abstract])) OR (Early Awakening[Title/Abstract])) OR (Awakening, Early[Title/Abstract])) OR (Sleep Initiation Dysfunction[Title/Abstract])) OR (Dysfunction, Sleep Initiation[Title/Abstract])) OR (Dysfunctions, Sleep Initiation[Title/Abstract])) OR (Sleep Initiation Dysfunctions[Title/Abstract])) OR (Sleeplessness[Title/Abstract])) OR (Insomnia[Title/Abstract])) OR (Insomnias[Title/Abstract]))) AND (("Cognitive Behavioral Therapy"[Mesh]) OR (((((((((((((((((((((((((((((((Cognitive behavioral therapy[Title/Abstract]) OR (CBT[Title/Abstract])) OR (Behavioral Therapies, Cognitive[Title/Abstract])) OR (Behavioral Therapy, Cognitive[Title/Abstract])) OR (Cognitive Behavioral Therapies[Title/Abstract])) OR (Therapies, Cognitive Behavioral[Title/Abstract])) OR (Therapy, Cognitive Behavioral[Title/Abstract])) OR (Psychotherapy, Cognitive[Title/Abstract])) OR (Therapy, Cognitive[Title/Abstract])) OR (Cognitive Therapies[Title/Abstract])) OR (Therapies, Cognitive[Title/Abstract])) OR (Cognitive Therapy[Title/Abstract])) OR (Cognitive Behaviour Therapy[Title/Abstract])) OR (Behaviour Therapies, Cognitive[Title/Abstract])) OR (Behaviour Therapy, Cognitive[Title/Abstract])) OR (Cognitive Behaviour Therapies[Title/Abstract])) OR (Therapies, Cognitive Behaviour[Title/Abstract])) OR (Therapy, Cognitive Behaviour[Title/Abstract])) OR (Cognitive Psychotherapy[Title/Abstract])) OR (Cognitive Psychotherapies[Title/Abstract])) OR (Psychotherapies, Cognitive[Title/Abstract])) OR (Cognition Therapy[Title/Abstract])) OR (Cognition Therapies[Title/Abstract])) OR (Therapies, Cognition[Title/Abstract])) OR (Therapy, Cognitive Behavior[Title/Abstract])) OR (Behavior Therapies, Cognitive[Title/Abstract])) OR (Cognitive Behavior Therapies[Title/Abstract])) OR (Therapies, Cognitive Behavior[Title/Abstract])) OR (Therapy, Cognition[Title/Abstract])) OR (Behavior Therapy, Cognitive[Title/Abstract])) OR (Cognitive Behavior Therapy[Title/Abstract])))) AND (("Mobile Applications"[Mesh]) OR ((((((((((((((((((((((((((((((Mobile Applications[Title/Abstract]) OR (Application, Mobile[Title/Abstract])) OR (Applications, Mobile[Title/Abstract])) OR (Mobile Application[Title/Abstract])) OR (Mobile Apps[Title/Abstract])) OR (App, Mobile[Title/Abstract])) OR (Apps, Mobile[Title/Abstract])) OR (Mobile App[Title/Abstract])) OR (Portable Software Apps[Title/Abstract])) OR (App, Portable Software[Title/Abstract])) OR (Portable Software App[Title/Abstract])) OR (Software App, Portable[Title/Abstract])) OR (Portable Software Applications[Title/Abstract])) OR (Application, Portable Software[Title/Abstract])) OR (Portable Software Application[Title/Abstract])) OR (Software Application, Portable[Title/Abstract])) OR (Portable Electronic Apps[Title/Abstract])) OR (App, Portable Electronic[Title/Abstract])) OR (Electronic App, Portable[Title/Abstract])) OR (Portable Electronic App[Title/Abstract])) OR (Portable Electronic Applications[Title/Abstract])) OR (Application, Portable Electronic[Title/Abstract])) OR (Electronic Application, Portable[Title/Abstract])) OR (Portable Electronic Application[Title/Abstract])) OR (smartphone[Title/Abstract])) OR (Smartphones[Title/Abstract])) OR (Smart Phones[Title/Abstract])) OR (Smart Phone[Title/Abstract])) OR (Phones, Smart[Title/Abstract])) OR (Digital[Title/Abstract])))) AND (("Depressive Disorder"[Mesh]) OR ((((((((((((((((((((((((((Depressive Disorder[Title/Abstract]) OR (Depressive Disorders[Title/Abstract])) OR (Disorder, Depressive[Title/Abstract])) OR (Disorders, Depressive[Title/Abstract])) OR (Neurosis, Depressive[Title/Abstract])) OR (Depressive Neuroses[Title/Abstract])) OR (Depressive Neurosis[Title/Abstract])) OR (Neuroses, Depressive[Title/Abstract])) OR (Depression, Endogenous[Title/Abstract])) OR (Depressions, Endogenous[Title/Abstract])) OR (Endogenous Depression[Title/Abstract])) OR (Endogenous Depressions[Title/Abstract])) OR (Depressive Syndrome[Title/Abstract])) OR (Depressive Syndromes[Title/Abstract])) OR (Syndrome, Depressive[Title/Abstract])) OR (Syndromes, Depressive[Title/Abstract])) OR (Depression, Neurotic[Title/Abstract])) OR (Depressions, Neurotic[Title/Abstract])) OR (Neurotic Depression[Title/Abstract])) OR (Neurotic Depressions[Title/Abstract])) OR (Melancholia[Title/Abstract])) OR (Melancholias[Title/Abstract])) OR (Unipolar Depression[Title/Abstract])) OR (Depression, Unipolar[Title/Abstract])) OR (Depressions, Unipolar[Title/Abstract])) OR (Unipolar Depressions[Title/Abstract]))) |
| **2.Cochrane** | |
| #1 | MeSH descriptor: [Sleep Initiation and Maintenance Disorders] explode all trees |
| #2 | (Sleep Initiation and Maintenance Disorders):ti,ab,kw OR (Disorders of Initiating and Maintaining Sleep):ti,ab,kw OR (Early Awakening):ti,ab,kw OR (Awakening, Early):ti,ab,kw OR (Sleep Initiation Dysfunction):ti,ab,kw |
| #3 | (Dysfunction, Sleep Initiation):ti,ab,kw OR (Dysfunctions, Sleep Initiation):ti,ab,kw OR (Sleep Initiation Dysfunctions):ti,ab,kw OR (Sleeplessness):ti,ab,kw OR (Insomnia):ti,ab,kw |
| #4 | (Insomnias):ti,ab,kw |
| #5 | #1 or #2 or #3 or #4 |
| #6 | MeSH descriptor: [Cognitive Behavioral Therapy] explode all trees |
| #7 | (Cognitive behavioral therapy):ti,ab,kw OR (CBT):ti,ab,kw OR (Behavioral Therapies, Cognitive):ti,ab,kw OR (Behavioral Therapy, Cognitive):ti,ab,kw OR (Cognitive Behavioral Therapies):ti,ab,kw |
| #8 | (Therapies, Cognitive Behavioral):ti,ab,kw OR (Therapy, Cognitive Behavioral):ti,ab,kw OR (Psychotherapy, Cognitive):ti,ab,kw OR (Therapy, Cognitive):ti,ab,kw OR (Cognitive Therapies):ti,ab,kw |
| #9 | (Therapies, Cognitive):ti,ab,kw OR (Cognitive Therapy):ti,ab,kw OR (Cognitive Behaviour Therapy):ti,ab,kw OR (Behaviour Therapies, Cognitive):ti,ab,kw OR (Behaviour Therapy, Cognitive):ti,ab,kw |
| #10 | (Cognitive Behaviour Therapies):ti,ab,kw OR (Therapies, Cognitive Behaviour):ti,ab,kw OR (Therapy, Cognitive Behaviour):ti,ab,kw OR (Cognitive Psychotherapy):ti,ab,kw OR (Cognitive Psychotherapies):ti,ab,kw |
| #11 | (Psychotherapies, Cognitive):ti,ab,kw OR (Cognition Therapy):ti,ab,kw OR (Cognition Therapies):ti,ab,kw OR (Therapies, Cognition):ti,ab,kw OR (Therapy, Cognitive Behavior):ti,ab,kw |
| #12 | (Behavior Therapies, Cognitive):ti,ab,kw OR (Cognitive Behavior Therapies):ti,ab,kw OR (Therapies, Cognitive Behavior):ti,ab,kw OR (Therapy, Cognition):ti,ab,kw OR (Behavior Therapy, Cognitive):ti,ab,kw |
| #13 | (Cognitive Behavior Therapy):ti,ab,kw |
| #14 | #6 or #7 or #8 or #9 or #10 or #11 or #12 or #13 |
| #15 | MeSH descriptor: [Mobile Applications] explode all trees |
| #16 | (Mobile Applications):ti,ab,kw OR (Application, Mobile):ti,ab,kw OR (Applications, Mobile):ti,ab,kw OR (Mobile Application):ti,ab,kw OR (Mobile Apps):ti,ab,kw |
| #17 | (App, Mobile):ti,ab,kw OR (Apps, Mobile):ti,ab,kw OR (Mobile App):ti,ab,kw OR (Portable Software Apps):ti,ab,kw OR (App, Portable Software):ti,ab,kw |
| #18 | (Portable Software App):ti,ab,kw OR (Software App, Portable):ti,ab,kw OR (Portable Software Applications):ti,ab,kw OR (Application, Portable Software):ti,ab,kw OR (Portable Software Application):ti,ab,kw |
| #19 | (Software Application, Portable):ti,ab,kw OR (Portable Electronic Apps):ti,ab,kw OR (App, Portable Electronic):ti,ab,kw OR (Electronic App, Portable):ti,ab,kw OR (Portable Electronic App):ti,ab,kw |
| #20 | (Portable Electronic Applications):ti,ab,kw OR (Application, Portable Electronic):ti,ab,kw OR (Electronic Application, Portable):ti,ab,kw OR (Portable Electronic Application):ti,ab,kw OR (smartphone):ti,ab,kw |
| #21 | (Smartphones):ti,ab,kw OR (Smart Phones):ti,ab,kw OR (Smart Phone):ti,ab,kw OR (Phones, Smart):ti,ab,kw OR (Digital):ti,ab,kw |
| #22 | #15 or #16 or #17 or #18 or #19 or #20 or #21 |
| #23 | MeSH descriptor: [Depressive Disorder] explode all trees |
| #24 | (Depressive Disorder):ti,ab,kw OR (Depressive Disorders):ti,ab,kw OR (Disorder, Depressive):ti,ab,kw OR (Disorders, Depressive):ti,ab,kw OR (Neurosis, Depressive):ti,ab,kw |
| #25 | (Depressive Neuroses):ti,ab,kw OR (Depressive Neurosis):ti,ab,kw OR (Neuroses, Depressive):ti,ab,kw OR (Depression, Endogenous):ti,ab,kw OR (Depressions, Endogenous):ti,ab,kw |
| #26 | (Endogenous Depression):ti,ab,kw AND (Endogenous Depressions):ti,ab,kw AND (Depressive Syndrome):ti,ab,kw AND (Depressive Syndromes):ti,ab,kw AND (Syndrome, Depressive):ti,ab,kw |
| #27 | (Syndromes, Depressive):ti,ab,kw OR (Depression, Neurotic):ti,ab,kw OR (Depressions, Neurotic):ti,ab,kw OR (Neurotic Depression):ti,ab,kw OR (Neurotic Depressions):ti,ab,kw |
| #28 | (Melancholia):ti,ab,kw OR (Melancholias):ti,ab,kw OR (Unipolar Depression):ti,ab,kw OR (Depression, Unipolar):ti,ab,kw OR (Depressions, Unipolar):ti,ab,kw |
| #29 | (Unipolar Depressions):ti,ab,kw |
| #30 | #23 or #24 or #25 or #26 or #27 or #28 or #29 |
| #31 | #5 and #14 and #22 and #30 |
| **3.Embase** | |
| #1 | 'insomnia'/exp |
| #2 | 'sleep initiation':ab,ti AND 'maintenance disorders':ab,ti OR ('disorders of initiating':ab,ti AND 'maintaining sleep':ab,ti) OR 'early awakening':ab,ti OR 'awakening, early':ab,ti OR 'sleep initiation dysfunction':ab,ti OR 'dysfunction, sleep initiation':ab,ti OR 'dysfunctions, sleep initiation':ab,ti OR 'sleep initiation dysfunctions':ab,ti OR sleeplessness:ab,ti OR insomnia:ab,ti OR insomnias:ab,ti |
| #3 | #1 OR #2 |
| #4 | 'cognitive behavioral therapy'/exp |
| #5 | 'cognitive behavioral therapy':ab,ti OR cbt:ab,ti OR 'behavioral therapies, cognitive':ab,ti OR 'behavioral therapy, cognitive':ab,ti OR 'cognitive behavioral therapies':ab,ti OR 'therapies, cognitive behavioral':ab,ti OR 'therapy, cognitive behavioral':ab,ti OR 'psychotherapy, cognitive':ab,ti OR 'therapy, cognitive':ab,ti OR 'cognitive therapies':ab,ti OR 'therapies, cognitive':ab,ti OR 'cognitive therapy':ab,ti OR 'cognitive behaviour therapy':ab,ti OR 'behaviour therapies, cognitive':ab,ti OR 'behaviour therapy, cognitive':ab,ti OR 'cognitive behaviour therapies':ab,ti OR 'therapies, cognitive behaviour':ab,ti OR 'therapy, cognitive behaviour':ab,ti OR 'cognitive psychotherapy':ab,ti OR 'cognitive psychotherapies':ab,ti OR 'psychotherapies, cognitive':ab,ti OR 'cognition therapy':ab,ti OR 'cognition therapies':ab,ti OR 'therapies, cognition':ab,ti OR 'therapy, cognitive behavior':ab,ti OR 'behavior therapies, cognitive':ab,ti OR 'cognitive behavior therapies':ab,ti OR 'therapies, cognitive behavior':ab,ti OR 'therapy, cognition':ab,ti OR 'behavior therapy, cognitive':ab,ti OR 'cognitive behavior therapy':ab,ti |
| #6 | #4 OR #5 |
| #7 | 'mobile application'/exp |
| #8 | 'mobile applications':ab,ti OR 'application, mobile':ab,ti OR 'applications, mobile':ab,ti OR 'mobile application':ab,ti OR 'mobile apps':ab,ti OR 'app, mobile':ab,ti OR 'apps, mobile':ab,ti OR 'mobile app':ab,ti OR 'portable software apps':ab,ti OR 'app, portable software':ab,ti OR 'portable software app':ab,ti OR 'software app, portable':ab,ti OR 'portable software applications':ab,ti OR 'application, portable software':ab,ti OR 'portable software application':ab,ti OR 'software application, portable':ab,ti OR 'portable electronic apps':ab,ti OR 'app, portable electronic':ab,ti OR 'electronic app, portable':ab,ti OR 'portable electronic app':ab,ti OR 'portable electronic applications':ab,ti OR 'application, portable electronic':ab,ti OR 'electronic application, portable':ab,ti OR 'portable electronic application':ab,ti OR smartphone:ab,ti OR smartphones:ab,ti OR 'smart phones':ab,ti OR 'smart phone':ab,ti OR 'phones, smart':ab,ti OR digital:ab,ti |
| #9 | #7 OR #8 |
| #10 | 'depression'/exp |
| #11 | depression:ab,ti OR 'depressive disorder':ab,ti OR 'depressive disorders':ab,ti OR 'disorder, depressive':ab,ti OR 'disorders, depressive':ab,ti OR 'neurosis, depressive':ab,ti OR 'depressive neuroses':ab,ti OR 'depressive neurosis':ab,ti OR 'neuroses, depressive':ab,ti OR 'depression, endogenous':ab,ti OR 'depressions, endogenous':ab,ti OR 'endogenous depression':ab,ti OR 'endogenous depressions':ab,ti OR 'depressive syndrome':ab,ti OR 'depressive syndromes':ab,ti OR 'syndrome, depressive':ab,ti OR 'syndromes, depressive':ab,ti OR 'depression, neurotic':ab,ti OR 'depressions, neurotic':ab,ti OR 'neurotic depression':ab,ti OR 'neurotic depressions':ab,ti OR melancholia:ab,ti OR melancholias:ab,ti OR 'unipolar depression':ab,ti OR 'depression, unipolar':ab,ti OR 'depressions, unipolar':ab,ti OR 'unipolar depressions':ab,ti |
| #12 | #10 OR #11 |
| #13 | #3 AND #6 AND #9 AND #12 |
| **4.Web of science** | |
| #1 | TS=(Sleep Initiation and Maintenance Disorders) OR TS=(Disorders of Initiating and Maintaining Sleep) OR TS=(Early Awakening) OR TS=(Awakening, Early) OR TS=(Sleep Initiation Dysfunction) OR TS=(Dysfunction, Sleep Initiation) OR TS=(Dysfunctions, Sleep Initiation) OR TS=(Sleep Initiation Dysfunctions) OR TS=(Sleeplessness) OR TS=(Insomnia) OR TS=(insomniacs) |
| #2 | TS=(Cognitive behavioral therapy) OR TS=(CBT) OR TS=(Behavioral Therapies, Cognitive) OR TS=(Behavioral Therapy, Cognitive) OR TS=(Cognitive Behavioral Therapies) OR TS=(Therapies, Cognitive Behavioral) OR TS=(Therapy, Cognitive Behavioral) OR TS=(Psychotherapy, Cognitive) OR TS=(Therapy, Cognitive) OR TS=(Cognitive Therapies) OR TS=(Therapies, Cognitive) OR TS=(Cognitive Therapy) OR TS=(Cognitive Behaviour Therapy) OR TS=(Behaviour Therapies, Cognitive) OR TS=(Behaviour Therapy, Cognitive) OR TS=(Cognitive Behaviour Therapies) OR TS=(Therapies, Cognitive Behaviour) OR TS=(Therapy, Cognitive Behaviour) OR TS=(Cognitive Psychotherapy) OR TS=(Cognitive Psychotherapies) OR TS=(Psychotherapies, Cognitive) OR TS=(Cognition Therapy) OR TS=(Cognition Therapies) OR TS=(Therapies, Cognition) OR TS=(Therapy, Cognitive Behavior) OR TS=(Behavior Therapies, Cognitive) OR TS=(Cognitive Behavior Therapies) OR TS=(Therapies, Cognitive Behavior) OR TS=(Therapy, Cognition) OR TS=(Behavior Therapy, Cognitive) OR TS=(Cognitive Behavior Therapy) |
| #3 | TS=(Mobile Applications) OR TS=(Application, Mobile) OR TS=(Applications, Mobile) OR TS=(Mobile Application) OR TS=(Mobile Apps) OR TS=(App, Mobile) OR TS=(Apps, Mobile) OR TS=(Mobile App) OR TS=(Portable Software Apps) OR TS=(App, Portable Software) OR TS=(Portable Software App) OR TS=(Software App, Portable) OR TS=(Portable Software Applications) OR TS=(Application, Portable Software) OR TS=(Portable Software Application) OR TS=(Software Application, Portable) OR TS=(Portable Electronic Apps) OR TS=(App, Portable Electronic) OR TS=(Electronic App, Portable) OR TS=(Portable Electronic App) OR TS=(Portable Electronic Applications) OR TS=(Application, Portable Electronic) OR TS=(Electronic Application, Portable) OR TS=(Portable Electronic Application) OR TS=(smartphone) OR TS=(Smartphones) OR TS=(Smart Phones) OR TS=(Smart Phone) OR TS=(Phones, Smart) OR TS=(Digital) |
| #4 | TS=(Depressive Disorder) OR TS=(Depressive Disorders) OR TS=(Disorder, Depressive) OR TS=(Disorders, Depressive) OR TS=(Neurosis, Depressive) OR TS=(Depressive Neuroses) OR TS=(Depressive Neurosis) OR TS=(Neuroses, Depressive) OR TS=(Depressive Syndrome) OR TS=(Depressive Syndromes) OR TS=(Syndrome, Depressive) OR TS=(Syndromes, Depressive) OR TS=(Melancholia) OR TS=(melancholics) OR TS=(Depression) OR TS=(Depressions)  #4 AND #3 AND #2 AND #1 |
