# Supplementary material for: The efficacy of digital cognitive behavioral therapy for insomnia and depression: a systematic review and meta-analysis of randomized controlled trials
Source: PeerJ. 2023 Oct 31;11:e16137. doi: 10.7717/peerj.16137 (PMC10624170; doi:10.7717/peerj.16137)
Supplement: Supplemental Information 7 [file peerj-11-16137-s007.docx]

## Systematic Review and/or Meta-Analysis Rationale

For systematic reviews / meta-analyses, authors need to provide the following information:

1.Comment: The rationale for conducting the systematic review / meta-analysis;

Responds: We would like to thanks for your comments. Insomnia is a complex and common disorder characterized by dissatisfaction with sleep quality or quantity due to difficulties in initiating and/or maintaining sleep. As a critical public health problem worldwide, insomnia frequently co-occurs with mental health disorders such as Major Depression and a risk factor for both incident and recurrent episodes. Meanwhile, people with insomnia had greater depression levels than people not having insomnia and were 9.82 times as likely to have clinically significant depression. Cognitive behavioral therapy for insomnia (CBT-I) is the recommended first-line therapy for patients with primary and comorbid insomnia. However, given the imbalanced geographical distributions of certified practices and the lack of qualified therapists, digitally-delivered CBT-I (dCBT-I) via web or mobile appears to be a more promising approach compared to traditional face-to-face CBT-I. However, RCTs on the efficacy of dCBT-I in alleviating depression are rare. The focus of this systematic review and meta-analysis is to highlight and critically review previous studies on dCBT-I for individuals with insomnia and depression, providing clinical references for the management of insomnia with comorbid depression.

2.Comment: The contribution that it makes to knowledge in light of previously published related reports, including other meta-analyses and systematic reviews.

Responds: Thank you very much for the comments. We included the most recent relevant studies to date for this systematic review and meta-analysis. Besides, we firstly assess the efficacy of dCBT-I in adults with insomnia and depression.
